# Secretory microRNA Profiles of Third- and Fourth-Stage *Dirofilaria immitis* Larvae with Different Macrocyclic Lactone Susceptibility: In Search of Biomarkers for Early Detection of Infection

**DOI:** 10.3390/pathogens10070786

**Published:** 2021-06-22

**Authors:** Lucienne Tritten, Erica J. Burkman, Tobias Clark, Guilherme G. Verocai

**Affiliations:** 1 Institute of Parasitology, University of Zurich, 8057 Zurich, Switzerland; 2 Department of Infectious Diseases, College of Veterinary Medicine, University of Georgia, Athens, GA 30602, USA; erica.burkman@boehringer-ingelheim.com; 3 Veterinary Medicine Research and Development, Zoetis Inc., 333 Portage Street, Kalamazoo, MI 49007, USA; tobias.clark@zoetis.com; 4 Department of Veterinary Pathobiology, College of Veterinary Medicine and Biomedical Sciences, Texas A&M University, College Station, TX 77843, USA

**Keywords:** diagnostic biomarkers, *Dirofilaria immitis*, heartworm, microRNA, early diagnosis, drug resistance, macrocyclic lactones, ivermectin

## Abstract

The canine heartworm, *Dirofilaria immitis*, is among the most important parasites of dogs in the United States and worldwide, and may cause severe and potentially fatal disease. Current diagnostic recommendations rely on serological detection of an adult female antigen, and visualization of microfilariae in the blood. Therefore, a reliable diagnosis can be only performed approximately six months post-infection. There is a growing need to characterize novel diagnostic markers that are capable of detecting the early stages of heartworm infection, in special markers associated with third-stage larvae (L3) and fourth-stage larvae (L4). The early detection of infection would guide medical interventions that could impede the development of patent infections and further parasite transmission. We cultured *D. immitis* L3 and L4 of two laboratorial strains with different susceptibility statuses to macrocyclic lactone drugs in vitro. Excretory/secretory microRNAs were sequenced and analyzed. We identified two miRNA novel candidates secreted abundantly by both L3 and L4 of both strains. These candidates were previously detected in the secretions of other *D. immitis* stages and one of them was found in the blood of *D. immitis*-infected dogs. These miRNAs have not been found in the secretions of other nematodes and could be *D. immitis*-specific diagnostic biomarkers, which could allow for the early detection of infection.

## 1. Introduction

The canine heartworm, *Dirofilaria immitis*, is arguably the most important parasite of dogs in the United States of America (USA) and across many countries worldwide. Heartworm is known to cause severe and often fatal disease in infected dogs, cats, and ferrets [1,2]. In addition to being the most prevalent filarial parasite of dogs in the country, heartworm incidence has been increasing in recent years, particularly in the southeastern United States [3].

The current guidelines of the American Heartworm Society recommend annual testing of all dogs in order to ensure the achievement and maintenance of successful prophylaxis. This recommendation includes the concurrent use of both an antigen detection test and a microscopy-based microfilariae (mf) detection test [4]. Currently, commercially available immunodiagnostic assays for the detection of heartworm antigens are used widely by practicing veterinarians and diagnostic laboratories. Such antigen detection tests target proteins secreted by mature adult *D. immitis* females. Despite their high sensitivity and specificity [4,5], a diagnosis can be only achieved approximately six months post-infection, which coincides with the average pre-patent period of heartworm.

There is a growing need to explore and characterize novel heartworm biomarkers that are capable of detecting the early stages of infection, that is, infections that are less than six months old. Earlier infections can be characterized by the presence of third-stage larvae (L3) and fourth-stage larvae (L4) and/or young adults (L5) within the organism of a definitive host, and the absence of sexually mature adult female and male worms in the hosts’ pulmonary arteries [6]. An early diagnosis of heartworm infection in conjunction with appropriate clinical management could be beneficial for the health and wellbeing of the infected individual and beyond by impeding the development of patent infections. As a consequence, fewer dogs would become reservoirs for infection of suitable Culicidae mosquito vectors, impacting heartworm transmission at a local scale. In addition, dogs would be less likely to progress into severe and/or fatal clinical heartworm disease.

Helminth parasites, particularly, have evolved sophisticated mechanisms of communication with their hosts. Various molecules secreted or excreted by helminths have been shown to participate in these processes [7,8]. Among them are microRNAs (miRNAs), short non-coding RNA molecules of 18–25 nucleotides in length, with important post-transcriptional regulatory functions of gene expression. The presence and detectability of nematode-specific miRNAs in hosts’ biofluids make them attractive diagnostic biomarker candidates for a wide range of diseases, and require minimal invasion [9]. To date, a handful of studies described the secreted miRNA profiles of various filarial nematodes, including *D. immitis* [9,10,11,12,13]. Such parasitic nematode-derived miRNAs have been found circulating in biological fluids (e.g., serum, plasma) and tissues of naturally infected hosts. The acknowledged presence of specific parasitic nematode-derived miRNAs in host serum and plasma highlights their potential as biomarkers of infection by filarioid species of veterinary and public health relevance [14,15].

Previously, *D. immitis*-derived miRNAs have been characterized in the plasma of dogs with patent experimental infections (therefore, with the concomitant presence of mf and adults) [9]. In addition, it was shown that some *D. immitis*-derived secretory miRNAs are differentially expressed across developmental stages or sexes of adults, highlighting their potential as markers for detecting the early stages of heartworm infection [16]. Additionally, nematode-derived miRNAs have been detected in the blood of animals and humans infected with tissue-dwelling filarial nematodes such as *Onchocerca volvulus*, *O. ochengi*, and *Loa loa*, which, similarly to *D. immitis* L3 and L4, do not reside in the circulatory system [10,12,17].

In order to assess the potential of *D. immitis*-derived miRNAs as diagnostic candidates in vivo, various elements remain to be clarified. In this study, we aimed to assess whether selected *D. immitis* L3 and/or L4-derived miRNAs in vitro may be biomarker candidates for the early detection of infection, both in terms of abundance and species specificity, as well as the consistency of stage-specific miRNA secretion across replicates and *D. immitis* laboratorial strains with different susceptibilities to macrocyclic lactone drugs. We compared our results to the existing literature and identified two novel miRNA candidates secreted abundantly by both L3 and L4 developmental stages and in both strains.

## 2. Results

### 2.1. Libraries and General Results

Six samples from each strain, Yazoo (resistant to macrocyclic lactones) and Missouri (susceptible to macrocyclic lactones) were analyzed, encompassing three replicates from L3, and three from L4. Libraries contained between 5.49 and 10.86 million reads. Across all samples (i.e., strains and stages), a total of 187 unique mature miRNAs with different degrees of confidence were discovered (Supplementary Table S1). For the subsequent analyses, we stratified the data by strain and/or stage (Supplementary Tables S2–S5).

### 2.2. Strain and Stage Specificity

We considered all miRNA candidates for our initial analysis, including those with no known orthologues among nematode sequences available in miRBase. We identified a total of 187 mature miRNA sequences across all six samples (Supplementary Table S1). The level of agreement between both strains and the two stages was high. Several members of the miR-100 family were among the top 10 most abundantly represented sequences, as well as lin-4 and let-7 (Table 1). Bma-miR-81, bma-miR-57, and bma-miR-92 were also among the highly abundant sequences present in all samples (if not among the top 10).

**Figure 1:**
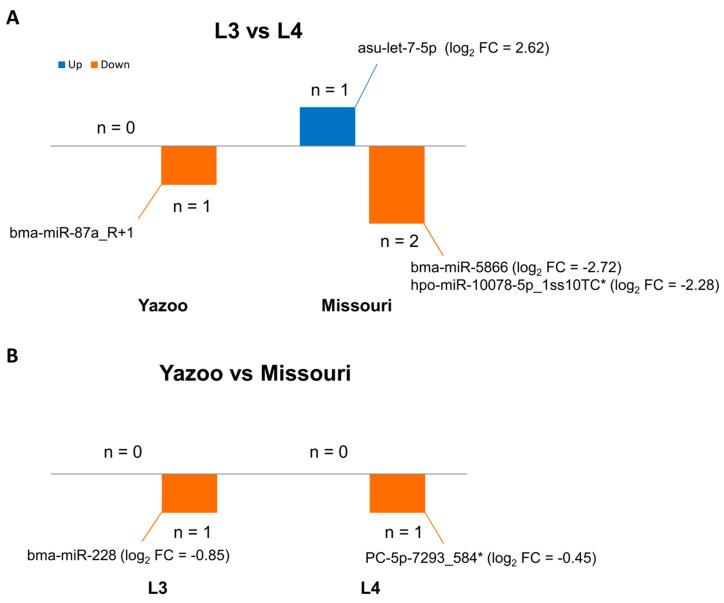
The number of differentially expressed miRNAs across pairwise comparisons. The differential expression was stratified by stages and strain. (**A**) Differentially expressed miRNAs between stages. For the Yazoo strain, bma-miR-87a_R+1 was absent in L3 secretions (hence, no fold-change). In the Missouri strain, asu-let-7-5p was more abundant in L3 compared to L4, and two sequences were less abundant in L3 compared to L4 (bma-miR-5866 and hpo-miR-10078-5p_1ss10TC). (**B**) Differentially expressed miRNAs between *D. immitis* strains. Bma-miR-228 was less abundant in Yazoo L3 (resistant to macrocyclic lactones) compared to Missouri L3 (susceptible to macrocyclic lactones); PC-5p-7293_584 was less abundant in Yazoo L4 compared to Missouri L4. * indicates ‘low confidence’ sequences, requiring validation. Log2 FC = log2 fold change in pairwise comparisons. All are statistically significant with a set *p*-value < 0.05.

Differential expression analysis was performed, taking all miRNAs into account expressed in at least one sample (Figure 1). One single miRNA (bma-miR-87a_R+1) was consistently found in L4 sample replicates in low levels (between 11 and 15 copies, normalized counts), but not in L3 samples of the strain Yazoo. The trend was similar in the strain Missouri, but the expression in both examined stages was not significantly different. bma-miR-87a_R+1 was always present in L4 secretions, found between 5 and 60 copies, and between 0 (in 2 samples) and 9 copies in L3 samples (Missouri, normalized counts). One miRNA (bma-miR-228) was found to be more abundant in Missouri than in Yazoo L3 products (normalized counts; Figure 1); it was, however, present in all replicates of both stages. Similarly, PC-5p-7293_584 was present in all samples and replicates were in high amounts and detected in slightly higher copy numbers in Missouri than in Yazoo L4 supernatants.

There were 2 predicted candidates (PC-5p-7293_584 and PC-3p-5433_825) that were also highly and consistently represented in all 4 samples (L3s and L4s of both strains) and are among the 20 most abundant miRNAs. These miRNAs map to the *D. immitis* genome but have no described orthologues among the available nematode miRNAs in miRBase v21. Hence, these might represent miRNAs specific to *D. immitis*. PC-5p-7293_584 was the 7th and 9th most abundant miRNA in L3 supernatants from the Yazoo and Missouri strains, respectively (191 and 393 copies), while it ranked 15th and 16th most abundant in L4 secretions, from the Yazoo and Missouri strains, respectively (>100 copies).

PC-3p-5433_825 was the 9th and 13th most abundant miRNA in L3 supernatants from the Yazoo and Missouri strains (>100 copies), respectively, while it ranked 10th and 12th most abundant in L4 from the Yazoo and Missouri strains, respectively (>200 copies); details are available in Supplementary Tables S2–S5.

### 2.3. Comparison to Previous Reports

**Figure 2:**
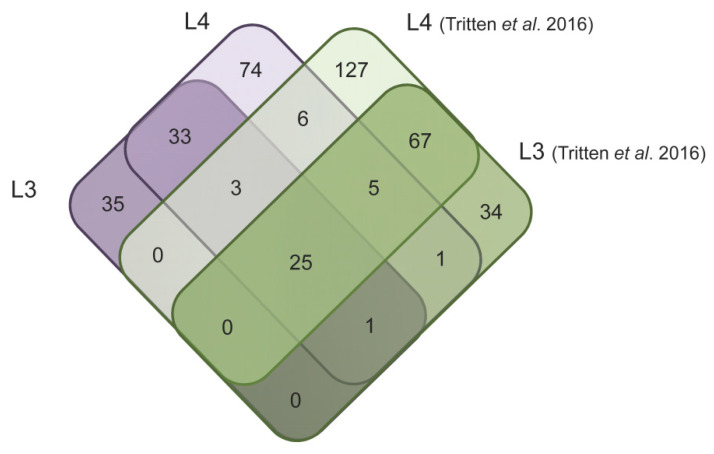
Venn diagram showing miRNAs common to datasets. Comparison of mature miRNA sequences across L3 and L4 secretions with a previous study [16]. Both strains of the current study were considered, as well as ‘low confidence’ sequences (e.g., not listed in miRBase), as they may include novel sequences.

At the sequence level, and taking all miRNAs into account regardless of the confidence status, 26 miRNAs were common to L3 secretions from the current study and an initial prior study conducted in vitro [16]. Similarly, 39 sequences were found in cultures from L4s in both studies, with both strains combined (Figure 2). In miRNA discovery experiments, it is likely that a fraction of predicted candidate sequences do not correspond to real miRNAs but rather to artifacts; therefore, these numbers must be interpreted with caution. Despite the fact that the same species and stages were examined in the compared studies, only a small fraction of miRNA sequences overlapped. Here, whole mature sequences were considered, despite the fact that the 3′ end of mature sequences are known to be subject to variation [18].

**Figure 3:**
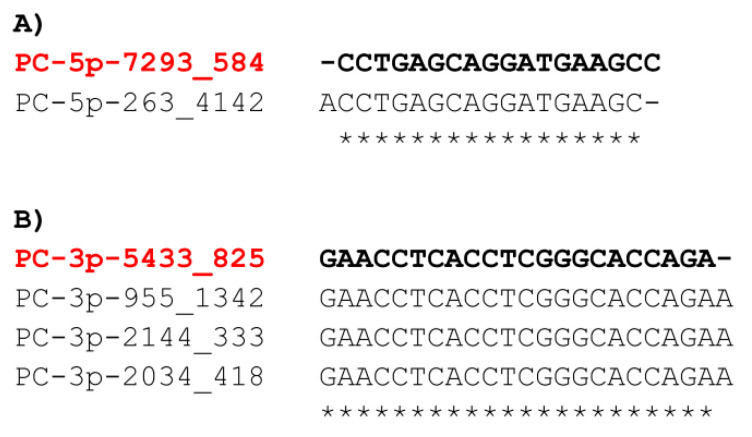
Sequence alignments of predicted candidates found in this study and previous reports. Sequences described in the current study are indicated in bold and red. (**A**) Alignments with PC-5p-7293_584. (**B**) Alignments with PC-3p-5433_825. Stars represent nucleotide matching positions in the predicted mature sequence. PC-5p-263_4142 and PC-3p-955_1342 were detected in vitro [16], while PC-3p-2144_333 and PC-3p-2034_418 were previously described in infected dogs (laboratory and natural infections) [9].

A very similar miRNA ranking by abundance had been shown in the supernatants of cultured L3 and L4, especially when extended beyond the top 10 shown in Table 1 [16]. A sequence similar to PC-5p-7293_584 was found in that previous report, under the name PC-5p-263_4142, and it was the 23rd and 32nd most abundant among L3 and L4 secretions, respectively. Similarly, PC-3p-5433_825 was found under the name PC-3p-955_1342 in the previous paper [16], and it was the 24th and 26th most abundant in L3 and L4 secretions, respectively. Both sequences were also released abundantly by mf, adult males, and females; however, PC-3p-5433_825 was slightly more abundant in the secretions of L3 and L4 in normalized count libraries (Table 2). However, these were not reported in the *D. immitis* somatic miRNAome [19], and have so far not been reported in the secretions of other filarial nematodes in vivo (in host blood). Sequence alignments with previously reported similar sequences are shown in Figure 3.

In *D. immitis*-infected dog serum, bma-miR-87a_R+1 was the 9th most abundant [9] and found in supernatants from all stages examined [16]. The sequence of PC-3p-5433_825 was found among the top 20 in dog serum as well (under the name PC-3p-2144_333), in a pool of samples from laboratory infections (from both Missouri and MP3 strains), as well as in a natural infection (under the name PC-3p-2034_418) [9] (Figure 3).

### 2.4. Target Prediction in Host Genome and Overrepresentation Analysis

In an attempt to understand the biological significance of the extracellular presence of these miRNAs in the canine host, computational target prediction within the dog transcriptome was conducted. Target prediction informs on the putative biological functions that *D. immitis* larvae may regulate in the dog by the secretion of miRNAs. Considering ‘high-confidence’ miRNAs (Groups 1, 2a, 2b; see Materials and Methods and Supplementary Table S1) and predicted candidates (Group 4), present in at least one sample in 10 copies or more, 58 L3 miRNAs and 94 L4 miRNAs were used for a computational target prediction within the dog genome, using the stand-alone TargetScan algorithm. Over 15,000 dog genes appeared to be targeted by the selected miRNAs from each stage. Overrepresentation analysis was used to identify the most significantly targeted host pathways within immune functions. The top 5 enriched pathways based on FDR are listed in Table 3. There were 84 categories significantly enriched in the predicted targets of L3 miRNAs. Behind several of the gene ontology (GO) descriptions are many fibroblast growth factor (FGF) isoforms, FGF receptors, as well as forkhead box proteins, among many others (146 in total). There were 64 categories significantly enriched in the predicted targets of L4 miRNAs. Generally, membrane transport features are predicted to be impacted by L4-secreted miRNAs. Interestingly, MAPK cascade was found among the top enriched categories.

## 3. Discussion

Our objective was to assess the consistency of the excretory/secretory miRNA profiles of L3 and L4 of two *D. immitis* strains in vitro, despite their different macrocylic lactone susceptibility status. Overall, we found a good agreement between the current study and previously published data for *D. immitis* [16], although there was generally an even higher agreement between L3 and L4 secretions from the same study, highlighting the impact of the inherent inter-experimental variation on resulting data. While the most abundant miRNA species overlapped substantially, their relative rank varied slightly. Regardless of the macrocyclic lactone susceptibility status of the two strains we have examined, our data suggest that the strain involved will have little impact on the most abundantly secreted miRNAs. There was not a dramatic impact of the two *D. immitis* laboratorial strains on the miRNA species we found, as only two miRNAs were significantly differentially expressed (bma-miR-228 and PC-5p-7293_584, by less than two-fold). Similarly, only four sequences appeared differentially expressed between L3- and L4-derived material (bma-miR-87a_R+1, bma-miR-5866, hpo-miR-10078-5p_1ss10TC, and asu-let-7-5p).

We have sequenced two candidate miRNA markers that are highly abundant and consistently found in both stages and both strains of *D. immitis* assessed in the study, but also that have been previously found in L3, L4, mf, and adults: PC-3p-5433_825 and PC-5p-7293_584. At the time of writing, these candidate markers did not have any match in miRBase [20,21] or MirGeneDB 2.0 [22], and hence, have no known mammalian orthologues. In addition, these sequences have not been detected in the secretions of other filarial nematodes in vivo thus far. Therefore, we believe that these miRNAs are potentially unique to *D. immitis*. The fact that these two predicted candidates have been found in the secretions of different *D. immitis* strains and across several independent studies speaks in favor of their actual existence (i.e., they are no artifacts). In addition, PC-3p-5433_825 was also discovered in the blood of heartworm-infected dogs, as well as in all *D. immitis* stages examined thus far [16]. The fact that both candidates were not detected in the somatic miRNA fraction may be due to the different computational approaches to search for novel miRNA candidates [19]. Both sequences were described in adult secretions in >100 copies; the sequence similar to PC-5p-7293_584 (PC-5p-263_4142) was detected abundantly in adult female supernatants (>1200 copies), however, with one base difference at the 5′ end.

Within the canine host, L3/L4-specific miRNAs may be detectable as early as 2 weeks post-infection when L3s are molting to L4 in the subcutaneous tissues. However, it is unknown if these would remain detectable after 60–70 days post-infection when most L4s should have molted to the young adult stage (L5) and may be already present in the pulmonary arteries and heart [6]. An important follow-up step will be to assess the temporal changes of *D. immitis*-derived miRNA profiles in the biofluids of experimentally infected dogs through the course of infection. This will allow us to start addressing potential overlaps or discrepancies between in vitro and in vivo secretions; it is currently unclear how well miRNA secretion profiles produced in vitro actually reflect in vivo ‘natural’ settings.

The detection of miRNA from L3 and L4 represents a challenge due to the small nematode biomass they represent relative to host tissues, their unresolved localization in the hosts’ organism, and their rapid development to the following developmental stages. Nevertheless, nematode-derived miRNAs of related filarioid nematodes whose neither adult nor larval stages reside in the hosts’ circulatory system have been detected in biofluids. This has been the case for tissue-dwelling filarial nematodes, *O. volvulus*, *O. ochengi*, and *L. loa* [11,12,13], all of which belong to the same family, Onchocercidae, as *D. immitis*. Circulating miRNAs are believed to be either stabilized in protein complexes or packaged in extracellular vesicles (e.g., in exosomes). In both cases, these miRNAs are highly stable and protected from enzymatic degradation or pH incompatibilities—a good characteristic for diagnostic biomarkers [23,24].

Lagatie and colleagues attempted to detect an array of *O. volvulus*-derived miRNAs in biofluids of naturally infected humans [25]. They concluded that circulating parasite miRNAs are not present at a sufficiently high level to be used as a biomarker for *O. volvulus* infection or treatment monitoring using LNA-based RT-qPCR analysis, as only some miRNAs showed detectable signals in some but not all samples [25]. While the analysis relied on a maximum volume of 200 µL serum or plasma, we argue that larger sample volumes may easily be acquired from dogs to be used for diagnosing *D. immitis* infections. In a previous study, several ml of blood were collected from mf-circulating mature *D. immitis* infections where dog serum had been processed for the detection of two *D. immitis*-specific miRNAs by RT-qPCR [9]. Custom-designed stem-loop RT-qPCR protocols may prove highly efficient to discriminate highly similar miRNA sequences and improve sensitivity [26]. While most studies on parasite-circulating miRNAs have focused on cell-free biofluids, it may be necessary to include the surrounding cell populations (such as phagocytes). In the *Litomosoides sigmodontis* mouse model, macrophages were shown to represent a parasite miRNA-enriched in vivo reservoir [27]. Generally, there is a need for comparative studies of miRNA profiles of different biofluids of dogs infected with *D. immitis*, as it could help to determine the usefulness of different sample types.

Many immune-related dog pathways are predicted to be targeted by the *D. immitis* L3 and L4 secreted array of miRNAs. These differ for L3 and L4 secretions and will be worth exploring further for insight into the interactions of larval stages with the canine host. Enriched GO terms resulting from L3 host target predictions revealed several pattern specification processes, which are broader networks involved in cell and organism differentiation and fate/commitment. Horizontal miRNA transfer from parasite to host has been shown; miRNA-driven gene downregulation may have an obvious impact on the host immune response among other fundamental biological processes [17,28,29]. Protein deubiquitination is a conserved mechanism to rescue proteins from degradation or to end ubiquitin signaling [30,31]. It is widely accepted that ubiquitination and deubiquitination are involved in various aspects of immune regulation, encompassing TCR signaling, T cell differentiation, immune tolerance, and so forth [32]. The accumulating evidence suggests potential interactions between so-called ‘immune checkpoint pathways’ and ubiquitination/deubiquitination in disease, including in the context of infectious diseases, for example, in bacterial or protozoal (*Trypanosoma*) infections [31,33]. Several members of the fibroblast growth factor (FGF) and FGF receptor family, among others, are potential targets of L3- and L4-derived miRNAs. These fulfill important roles in cell signaling and in regulating the expression of genes involved in cell growth, proliferation, differentiation, and longevity [34]. MAPKs are involved in various cellular responses, including the production of proinflammatory cytokines or antigen presentation. In helminth infections, signaling through toll-like receptors is mediated through the MAPK cascade; MAPK pathway and ERK1/2 activation support the induction of regulatory T cells/T helper 2 induction [8]. Some parasitic nematode-derived proteins, such as the *Acanthocheilonema viteae* cystatin have been shown to act as negative regulators of MAPK via other molecules, inducing regulatory responses [35]. Similarly, many bacterial pathogens have been shown to target crucial branches of the MAPK signaling in order to put pressure on the host [36]. Overall, upon uptake by host cells, *D. immitis*-derived miRNAs have the potential to modulate host immune responses, according to the current computational predictions. These would require experimental validation of the predicted targets in order to represent conclusive results.

## 4. Materials and Methods

### 4.1. Mosquito Rearing

For each mosquito cycle, only quality mosquito eggs of *Aedes aegypti* (Liverpool strain) were selected to propagate consequent mosquito cycles in an environmental chamber. The mosquito larvae were fed larvae food daily until pupation. The pupae were sorted by sex, discarding roughly 80% of the males; the majority of the female pupae were placed into rearing buckets.

### 4.2. D. immitis Infection for Obtaining L3s and L4s

The adult female mosquitoes were fed *D. immitis*-infected blood for a minimum of 2.5 h. The mosquitoes were dissected for L3 collection 14 days after blood feeding. The 2 *D. immitis* strains used were Missouri-2005 (susceptible to macrocyclic lactones) and Yazoo-2013 (resistant to macrocyclic lactones) [37,38]. These 2 strains were chosen to represent the different macrocyclic lactone susceptibility and were based on their availability in the laboratory.

### 4.3. In Vitro Culturing of L3s and L4s

Batches of approximately 250 *D. immitis* L3s were washed a minimum of five times with fresh RPMI to completely remove mosquito debris. Fresh culture media was added to well plates and L3s were added into trans-well plates and incubated in a 5% CO_2_ incubator at 37 °C. The supernatant was collected every 48 h for two weeks. The supernatant collected on days 2 and 4 of culture were designated as L3-derived supernatants; those collected on days 8 and 12 were L4-derived supernatants. After each supernatant collection, dead L3/L4s were removed and new sterile media were added to the remaining live worms. In total, there were 3 experimental replicates per larval stage and strain (Table 4). All supernatant samples were stored at −80 °C until further processing.

### 4.4. RNA Extraction

Total RNA was extracted from L3- and L4-derived supernatant of each strain, in triplicate, using the Norgen plasma/serum RNA purification midi kit (Norgen Biotek Corp., Thorold, ON, Canada) [16]. The RNA was extracted from 1 mL of each supernatant sample, eluted in 25 µL, and stored at −80 °C. To avoid cross-contamination, the supernatants of 2 different strains were never extracted alongside each other.

### 4.5. RNA Sequencing and Analysis

There were 6 samples from each strain, encompassing 3 replicates from L3 and 3 from L4 (Table 4), submitted to miRNA deep sequencing. The sequence input for sequencing was limited to the 18–30 nt size range. FastQC comprised a per-base sequence quality assessment for each submitted sample (not shown). The general characteristics of the reads (e.g., read length distribution) obtained for the different libraries were assessed by LC Sciences. The raw data were filtered using a filter module in an in-house program to delete low-quality reads, 3’ adapter sequences, and contaminations. The sequences ≥18 nt of clean data were annotated in the Rfam database (http://www.sanger.ac.uk/software/Rfam) [39] to remove non-coding RNA (rRNA, tRNA, snRNA, snoRNA) and degradation fragments of mRNA. The remaining sequences were aligned against all nematode miRNAs listed in miRbase (Release 21, http://www.miRbase.org) [20,21]; perfectly matched sequences were considered conserved *D. immitis* miRNAs. Nematode species, for which miRNAs are available in miRNAse, are the following: *Brugia malayi*, *Ascaris suum*, *Haemonchus contortus*, *Heligmosomoides polygyrus*, *Strongyloides ratti*, *Caenorhabditis elegans*, *C. briggsae*, *C. brenneri*, *C. remanei*, *Pristionchus pacificus*, and *Panagrellus redivivus*. Reads were then assigned to groups reflecting the confidence level that these sequences are truly *D. immitis* miRNAs. These groups are based on genome mapping properties, secondary structure, and homology to known miRNAs. Group 1: the reads were mapped to miRNAs/pre-miRNAs of specific species in miRbase, and the pre-miRNAs were further mapped to a genome and EST; Group 2a: the reads were mapped to miRNAs/pre-miRNAs of selected species in miRbase, and the mapped pre-miRNAs were not further mapped to a genome, but the reads (and, of course, the miRNAs of the pre-miRNAs) were mapped to a genome. The extended genome sequences from the genome loci may form hairpins; Group 2b: the reads were mapped to miRNAs/pre-miRNAs of selected species in miRbase and the mapped pre-miRNAs were not further mapped to a genome, but the reads (and, of course, the miRNAs of the pre-miRNAs) were mapped to a genome. The extended genome sequences from the genome loci may not form hairpins; Group 3: the reads were mapped to miRNAs/pre-miRNAs of selected species in miRbase and the mapped pre-miRNAs were not further mapped to a genome, and the reads were not mapped to a genome, either, but the reads were mapped to the mature miRNAs; Group 4 (predicted candidates): the reads were not mapped to pre-miRNAs of selected species in miRbase, but the reads were mapped to a genome, and the extended genome sequences from the genome may form hairpins. The normalization of sequence counts from each sample (or dataset) was achieved by dividing the counts by a library size parameter of the corresponding sample. The library size parameter is a median value of the ratio between the counts of a specific sample and a pseudo-reference sample. A count number in the pseudo-reference sample is the count geometric mean across all samples (see [16]).

### 4.6. Predicted miRNA Targets and Pathway Enrichment

miRNA target prediction was performed using the standalone TargetScan software package v. 7 (http://www.targetscan.org) [40], implemented as a standalone workflow under iPortal [41] and openBIS [42], using default parameters. All host genes that were targeted at least twice, regardless of the context scores, were used in enrichment analyses. The R version of the WEB-based Gene SeT Analysis Toolkit (WebGestalt) [43,44] was employed for overrepresentation analysis (ORA) based on gene ontology “biological processes” [45], using default parameters, with FDR < 0.05. As a reference gene list, we employed the immunological signatures (collection C7) from the Molecular Signatures Database v 6.2 (Gene Set Enrichment Analysis database, http://software.broadinstitute.org/gsea/msigdb/index.jsp) [46,47].

## 5. Conclusions

Mature miRNA sequences shed by L3 and L4 overlapped largely, both qualitatively and quantitatively. Similarly, the laboratorial *D. immitis* strain did not significantly impact the excretory/secretory miRNA profile in vitro, regardless of their macrocyclic lactone susceptibility status. The presence and temporal expression of the candidate miRNA biomarkers in vivo in biofluids of the canine host should be further investigated. The potential of L3- and L4-specific sequences as biomarkers of early heartworm infection remains to be assessed and validated in vivo using samples derived from experimentally and naturally infected dogs. Nevertheless, there are challenges associated with the limited biomass of such larval stages and sensitivity issues of the currently available miRNA detection assays.

## Figures and Tables

**Table 1:** Top 10 most abundant miRNAs. The ranking was established based on differential expression analysis for Yazoo and Missouri separately.

Top 10 Most Abundant miRNAs					Previous Study [16]	
	Yazoo L3	Missouri L3	Yazoo L4	Missouri L4	L3	L4
1	bma-miR-100a_R+1 **	bma-miR-100a_R+1 **	bma-miR-100a_R+1 **	bma-miR-100a_R+1 **	bma-lin-4	bma-lin-4
2	bma-miR-100b_R+2 *	bma-miR-100b_R+2 *	bma-lin-4 *	bma-lin-4 **	PC-3p-389_2939	bma-miR-92_R+2
3	bma-lin-4 *	bma-lin-4 *	bma-miR-100b_R+2 *	bma-miR-100b_R+2 *	asu-let-7-5p	asu-let-7-5p
4	bma-miR-100d_R+1 *	bma-miR-100d_R+1 *	bma-miR-100d_R+1 *	bma-miR-100d_R+1 *	bma-miR-100b_R+1	bma-miR-100a_R+1
5	asu-let-7-5p	asu-let-7-5p	bma-miR-81_R+1	bma-miR-81_R+1 *	bma-miR-100a_R+1	bma-miR-100b_R+1
6	bma-miR-100c_R+1_1ss12CT	bma-miR-81_R+1	bma-miR-100c_R+1_1ss12CT	bma-miR-57	bma-miR-92_R+2	bma-miR-57
7	**PC-5p-7293_584**	bma-miR-100c_R+1_1ss12CT	bma-miR-57	bma-miR-92_R+2	bma-miR-100d_R+1	bma-miR-100d_R+1
8	bma-miR-57	bma-miR-57	bma-miR-92_R+2	bma-miR-100c_R+1_1ss12CT	bma-miR-5364	PC-3p-389_2939
9	**PC-3p-5433_825**	**PC-5p-7293_584**	asu-let-7-5p	bma-miR-239-5p	bma-miR-228	bma-miR-5364
10	bma-miR-81_R+1	bma-miR-7_R+1_1ss10TA	**PC-3p-5433_825**	bma-miR-71_R+4	hco-miR-9	bma-bantam_R+1 (= bma-miR-81_R+1)

** present in >10,000 copies (normalized counts); * present in >1000 copies (normalized counts). ‘Predicted candidates’ of interest are bolded.

**Table 2:** Literature reportings of two predicted candidates in *D. immitis* and other filarial nematodes. Other filarial species included *Onchocerca ochengi*, *Loa loa*, *O. volvulus*, and *Litomosoides sigmodontis* * (studies based on host blood; [9,10,12,17]).

	In Supernatants? [16]	In Infected Dog Blood? [9]	In *D. immitis* Somatic miRNA Pool? [19]	In Other Filarial Species? *
PC-5p-7293_584	Yes	No	No	No
PC-3p-5433_825	Yes	Yes	No	No

**Table 3:** Overrepresentation analysis (gene ontology, biological processes) of predicted dog targets of *D. immitis* L3 and L4-derived miRNAs.

***D. immitis* L3 Gene Ontology—Biological Processes**			
**Gene Set**	**Description**	***p*-Value**	**FDR**
GO:0007389	pattern specification process	5.30 × 10^−8^	0
GO:0016579	protein de-ubiquitination	6.47 × 10^−8^	0
GO:0000122	negative regulation of transcription by RNA polymerase II	8.96 × 10^−8^	0
GO:0009952	anterior/posterior pattern specification	9.44 × 10^−8^	0
GO:0045934	negative regulation of nucleobase-containing compound metabolic process	1.94 × 10^−7^	0
***D. immitis* L4 Gene Ontology—Biological Processes**			
**Gene Set**	**Description**	***p*-Value**	**FDR**
GO:0015672	monovalent inorganic cation transport	4.65 × 10^−8^	0
GO:0098655	cation transmembrane transport	7.67 × 10^−8^	0
GO:0098662	inorganic cation transmembrane transport	7.84 × 10^−8^	0
GO:0000165	MAPK cascade	1.26 × 10^−7^	0
GO:0071804	cellular potassium ion transport	2.67× 10^−11^	4.45 × 10^−8^

**Table 4:** Experimental replicates and cultured larvae.

Experimental Replicate	*D. immitis* Strain	Number of L3 Cultured
1	Yazoo	3640
2	Yazoo	1560
3	Yazoo	1560
4	Missouri	3120
5	Missouri	1560
6	Missouri	780

## Data Availability

The raw data reads were deposited in Gene Expression Omnibus (GEO; http://www.ncbi.nlm.nih.gov/geo/) under the accession number GSE174744.

## Supplementary Materials

The following are available online at https://www.mdpi.com/article/10.3390/pathogens10070786/s1: Table S1. miRNAs shed by two *D. immitis* strains, L3 and L4 stages in vitro; Table S2. miRNAs shed by *D. immitis* L3 in vitro (stratified by strain); Table S3. miRNAs shed by *D. immitis* L4 in vitro (stratified by strain); Table S4. miRNAs shed by *D. immitis* strain Yazoo in vitro (stratified by stage); Table S5. miRNAs shed by *D. immitis* strain Missouri in vitro (stratified by stage).
